# Supplementary material for: Are carbon emissions trading and green financial instruments synergistic? -Comprehensive quantitative research based on content analysis
Source: PLoS One. 2024 Mar 7;19(3):e0298601. doi: 10.1371/journal.pone.0298601 (PMC10919601; doi:10.1371/journal.pone.0298601)
Supplement: S1 Data — (ZIP) [file pone.0298601.s001.zip › Data/地级市Z_F工作报告.zip!/╣π╬≈/░┘╔1⁄2╩╨/└┤╫╘╣1⁄2╓┌║┼í╛┬φ┐╦╩2╛▌═°í┐_2019.docx]

一、2018年工作回顾

　　我们坚持以习近平新时代中国特色社会主义思想为指导，认真贯彻落实中央、自治区和市委决策部署，积极应对复杂严峻的国内外形势，攻坚克难，持续推进稳增长、促改革、调结构、惠民生、防风险工作，经济社会总体平稳、稳中提质、稳中向好，地区生产总值增速继续保持高于全国、全区的增长速度。

　　预计，地区生产总值增长9%左右。财政收入增长8%。规模以上工业增加值增长12%。社会消费品零售总额增长10%。外贸进出口总额增长11 %。城镇居民人均可支配收入增长5.3 %，农村居民人均可支配收入增长10%，生态环境保持优良，民生事业持续改善。较好完成了四届人大三次会议提出的目标任务，各项工作取得了不少新突破、呈现了不少新亮点。

　　——成功举办百色庆祝自治区成立60周年活动，圆满完成中央代表团一分团到百色走访慰问各项任务。

　　——右江区成功脱贫摘帽，田阳、田东、平果、西林4个县有望脱贫摘帽，226个贫困村、20.2万贫困人口有望脱贫。

　　——深化领导班子建设年和投资环境整治年，投资环境满意度84.2，连续两年排全区第4位，创下2006年以来最好成绩。

　　 ——“铝二次创业”全面发力，百色区域电网总装机容量232万千瓦，电解铝产量150万吨。自治区政府将百色铝产业列为全区专项工作来推进。

　　——全国深度贫困地区高质量脱贫研讨会、全国工商联扶贫工作会、全国第二届生态有机产业创新发展大会、全区油茶产业发展现场会、全区易地扶贫搬迁工作现场会、全区健康扶贫现场会、全区就业扶贫现场推介会、全区屯级道路建设工作现场会、全区扶贫干部项目管理现场交流培训班、全区深化统计管理体制改革经验交流会等全国、全区重大会议在百色召开。

　　——开通百色至北京航线，拉近了老区百色与首都北京的距离，实现了对全国直辖市、长三角、珠三角等国内一线城市的航线覆盖。

　　——百色至河池、靖西至龙邦高速公路建成通车，“百色一号”开通中欧班列、中越跨境直通班列，互联互通水平进一步提升。

　　——百色水库灌区项目获得批复并开工建设，总投资41亿元，是百色撤地设市以来国家投资的最大项目。

　　——百东新区成功列入4个自治区级重点城市新区，“再建一座百色新城”迎来重大发展机遇！

　　一年来，我们敢于担当，主动作为，扎实推进各项工作。

　　（一）推进铝制造业提速提质，工业高质量发展初见成效。“铝二次创业”持续推进，电解铝产量150万吨，铝材加工308万吨，铝及配套产业产值达1280亿元。百矿新山年产30万吨、华磊新材料年产40万吨电解铝项目满产运行，百矿德保、百矿田林、新山苏源等项目一期投产。福地平铝80万吨再生铝项目开工建设，成为全国最大的再生铝项目。天桂铝业、百矿那坡等氧化铝项目有序推进。百矿高纯铝、平铝铝家具、靖西鲁桂等一批铝精深加工项目建成投产。百矿高新铝业、润泰铝业、百兴金兰铝板带箔等项目开工建设。铝深加工向铝镁合金线、汽车铝部件等高附加值方向发展。强强碳素预焙阳极、和泰科技氟化铝、中铝50万吨赤泥综合利用项目建成投产。百矿田东、百矿田林共100万吨碳素项目开工。兴义至百色输电线路、靖西湖润、德保安阳等输变电工程竣工，瓦村水电站首台机组、华磊3×35万千瓦机组并网发电，工业用电量增长49.5%。冶金、建材等传统产业稳步增长。华银铝业、华润水泥荣获第四届自治区主席质量奖提名奖。新增13个广西名牌产品。8个产品获批国家低碳产品认证。百矿铝业、凌云浪伏茶业获评广西工业企业质量管理标杆，平果双迎门业被认定为自治区级企业技术中心，银海铝业等获评广西智能工厂示范企业。

　　（二）加快农业农村发展，乡村振兴开局良好。粮食、蔬菜等大宗农产品供给稳固。芒果、油茶等5个百万亩工程持续推进，百色芒果的全国影响力进一步提升，油茶种植规模全区第一。建成国家级“一村一品”示范村13个、自治区级5个，现代特色农业示范区248个。新建富硒基地10个，新增无公害产品18个，新认证有机农产品12个，有机农产品基地认证规模全区第一。“百色山茶油”获得地理标志证明商标。“田林灵芝”“靖西大果山楂”“凌云乌鸡”获得农产品地理标志登记认证，国家“地标产品”认定总量全区第一。累计通过自治区生态养殖场认证222个，新增规模养殖场126个，生态养殖场认证率全区第一。乐业获批国家有机产品认证示范创建区。田东获百色芒果广西特色农产品优势区认证。新增农民专业合作社569家、家庭农场100家。推进美丽乡村建设，投入资金46亿元，饮水、用电、通信、照明等基础设施持续改善。957个村集体经济年收入3万元以上，1798个行政村全部建成“六有”标准综合服务中心。开工建设通屯道路1593条。特色村寨和传统村落保护力度加大。成功举办广西首届“中国农民丰收节”。田阳县美丽乡村建设获批国家第三批农村综合改革标准化试点。

　　（三）优化新产业新业态布局，现代服务业不断壮大。设立服务业发展专项资金。一批服务业企业入驻新山、龙邦服务业集聚区。新增限上规上服务业企业120家，排全区第三。新增广西服务业品牌3个。数字产业加快布局，签约科大讯飞等4家企业。建成凌云浪伏小镇、乐业故事小镇等健康养生基地。田东县被评为广西健康产业发展示范县。“百色一号”专列全年开行47趟，累计运输果蔬产品、海产品2.6万吨。建成电子商务市县两级运营服务中心11个。启动创建国家全域旅游示范区。新增百色欢乐小镇等AAAA级景区5家，百色起义纪念园创AAAAA级景区通过自治区初评。靖西、乐业成功创建广西特色旅游名县，实现零的突破。旅游接待总人数增长29%，旅游总消费增长36%。新增文化产业示范基地1家。金融、房地产业稳步发展，金融机构各项存贷款余额增长7.5%。全市商品房销售面积增长23%。

　　（四）持续推进精准脱贫，脱贫质量不断提升。打好脱贫攻坚“六场硬仗”，投入财政扶贫资金69.65亿元，统筹整合各类涉农资金36.23亿元。完成向1854个行政村（社区）选派第一书记和扶贫工作全覆盖。完成公路建设3500公里，改造农村危房7928户、农村饮水安全工程2282处。“5+2”特色产业贫困户覆盖率达93.71%。贫困劳动力累计就业20.7万人。易地扶贫搬迁累计完成17.45万人，占“十三五”计划总数的96.4%。深圳小镇一期竣工，2315户贫困户9831人入住，成为粤桂扶贫协作新亮点。健康扶贫“五道医疗保障线”全面形成，在全区率先成立健康扶贫“一站式”服务工作站，贫困患者住院费实际报销比例连续两年在全区率先达到90%以上。发放教育资助补助金7.1亿元，受益学生127.97万人次。金融机构累计投放扶贫贷款147亿元，8.46万贫困户获得扶贫小额信贷。16.59万贫困人口纳入农村低保。生态扶贫投入资金1.26亿元，受益贫困户8.92万户。816个贫困村集体经济收入超过3万元。“爱心公益超市”“扶贫车间”建设获自治区高度肯定。

　　（五）打好蓝天碧水净土保卫战，生态环境质量不断改善。PM2.5平均浓度36.6微克/立方米，下降12.9%，空气质量优良率达93.7%，提升3.6%。万峰湖网箱养殖清理、下雷河综合整治成效显著，4个工业区污水集中处理设施建设完成。地表水考核断面水质优良率、市县两级饮用水源地水质达标率100%。开展土壤污染治理与生态修复，全市涉危险废物企业规范化管理达到年度考核目标。完成水土流失治理526平方公里。植树造林58.89万亩，造林总面积连续八年排全区第一，森林覆盖率达78.24%。推进“山水林田湖草”生态保护和修复试点项目。完成中央环保督察“回头看”移交问题整改104件。凌云县荣获国家生态文明建设示范县称号。平果县采矿临时用地的创新做法获得国务院通报表扬。国家气候适应型试点城市建设加快实施。中铝广西分公司被认定为国家绿色工厂示范企业。

　　（六）积极化解重点领域风险，金融保障不断强化。银行业金融服务体系不断完善，农村信用体系“四级联创”各项指标名列全区首位。98家市场主体、9家金融机构56个网点开办跨境结算业务。银企对接为企业融资136.5亿元。开展“中小微企业金融服务促进年”活动，为698家企业降低融资成本约1亿元。成功发行全国首单地市级扶贫资产证券化产品1.8亿元。开展政府隐性债务清查，全市政府性债务风险总体可控。加强非法集资活动的监测预警和打击，开展互联网金融风险专项整治。

　　（七）强化重大项目建设，支撑发展更加有力。一批重大项目开竣工，市层面统筹推进重大项目521项，完成投资约400亿元，开工项目90个，竣工项目60个。百色机场新增4条客运航线，航空货运站建成投入使用。乐业通用机场选址获空军总部核准。田林瓦村水电站竣工。田林至西林高速公路、百色大道三期项目、凤山至乐业公路开工建设。乐百高速、万生隆国际商贸物流项目等自治区重大项目进展加快。推进市本级9个重点PPP项目建设，总投资329.58亿元。

　　（八）加快推进产城融合，城镇化大格局逐步形成。《百色市城市总体规划（2017年~2035年）》获自治区批复。百东新区核心规划面积由29平方公里调整为50平方公里，深百产业园、市人民医院百东分院、右江民族医学院百东校区、体育中心二期等项目加快推进。国家卫生城市成果不断巩固。田阳撤县设区、平果撤县设市进一步加快。平果新安工业小镇、田阳百育芒果小镇和田东祥周商贸物流小镇列为广西第一批特色小镇。全市建成区面积预计达155平方公里，常住人口城镇化率达38%。

　　（九）不断推动对外开放，开放合作呈现新局面。《广西百色重点开发开放试验区建设实施方案》上报国家发改委。龙邦、平孟、岳圩、湖润四个边贸产业园区开工建设。跨境劳务合作取得新突破。“百色一号”对接中欧班列常态化运营，开通中越集装箱直通班列。与泛珠三角地区、粤港澳大湾区、长三角等地区企业签约项目61个。左右江革命老区振兴规划深入实施，储备新的重大项目428个。跨省（区）经济合作园区建设扎实推进，西林—广南产业园区启动建设。招商引资区外到位资金468.37亿元，增长3.6%。

　　（十）深化改革创新，发展活力进一步释放。供给侧结构性改革深入推进，淘汰铁合金产能1.11万吨，化解煤矿过剩产能9万吨。区域电网下调电价0.16元，累计降电费24亿元。54家重点中小企业通过“惠企贷”获得贷款3.87亿元。落实减税降费政策，减免税收26亿元。“放管服”改革承接、调整、取消行政审批事项72项。机构改革方案已上报自治区，国家税收征管体制改革完成市县国税地税机构合并。商事制度改革实现市场主体登记管理全程电子化。完成自治区统计管理体制改革试点。文化、教育、卫生、水利、国有林场等体制改革取得新成效。完成10家国有企业整合重组，百矿集团成为广西唯一入选国家“双百行动”改革的市级国有企业。推进沿边金融改革，跨境人民币业务结算累计达61亿元。完成农村“两权”抵押贷款试点任务，贷款余额增至3.45亿元。在全区率先推出不动产抵押登记向银行延伸和免继承公证登记。城乡建设用地增减挂钩总规模4.2万亩，排全区第二。盘活存量用地2.77万亩，供地率排名全区第二。百色高新区创建国家级高新区上报国务院。建成广西院士工作站1个，实现零的突破。国家人社部批准百矿集团成立博士后工作站。出台“1+6”人才引进政策，招聘重点领域高层次人才263人。获国家认定“星创天地”5家。新增高新技术企业12家。在全区首创线上线下“众包一站式”质量技术服务模式。全市发明专利授权增长78.69%，增速排全区第1位。

　　（十一）持续保障和改善民生，群众获得感幸福感安全感不断增强。全市用于民生方面支出321.32亿元，占一般公共预算支出比重82%。筹措78亿元实施社保、健康、教育等十大类惠民工程。新增城镇就业2.2万人，城镇登记失业率2.27%。新增转移农村劳动力7.56万人次，完成164.83%。社会保险参保总人数达675.28万人次，增长2.39%。参加城乡居民基本养老保险180.88万人次，发放养老金51.38万人次，发放率100%。全年累计发放低保金8.8亿元。城乡低保补助由月人均330元、170元分别提高到350元、190元。累计发放特困人员、边民、残疾人、困难群众、高龄老人、优抚对象等政策性补贴补助6.35亿元。建成村级公共服务中心152个。新一轮三年棚户区改造规模全区第一，开工建设棚改房1.8万套，建成3219套。完成农村改厕改厨任务各9.8万户。实施“全面改薄”等各类教育项目2105个，乡镇公办幼儿园基本实现全覆盖。右江区、平果、那坡、乐业、西林义务教育均衡发展通过国家督导评估。艰苦边远地区乡村教师待遇保障水平不断提高，招聘中小学、幼儿园教师3208名，乡村教师“难招难留”得到缓解。实施基层卫生基建项目160个。城区卫生项目建设攻坚战开工9个，市人民医院综合大楼、市妇保院公共卫生服务楼等项目基本完工，百色市食品药品检验大楼竣工验收。

　　百色市博物馆、群众艺术馆、城市规划展示馆、青少年活动中心项目等公共文化服务项目加快建设。百胜街、解放街获评第二批自治区级历史文化街区。打造一批文化艺术精品，自治区成立60周年大庆唯一一部献礼电影《又是一年三月三》在北京人民大会堂首映后在全国院线放映。扶贫微电影《心愿》荣获第六届亚洲微电影“金海棠奖”优秀作品奖。田林壮剧《祭瑶娘》荣获第十届广西壮剧展演“桂花奖”。《百色市志》编纂完成。全市第五届文艺汇演圆满完成。竞技体育水平不断提高，我市运动员共获得全国各类赛事金牌42枚。成功举办第五届全市运动会、“丝路杯”中国—东盟乒乓球赛、红城百色国际半程马拉松赛、全国徒步大会百色“地心之旅”等重大赛事，竞技体育与全民健身深度融合。

　　社会治理不断加强。推动安全生产领域改革政策落地生根，开展重点行业领域安全生产专项整治行动，全市安全生产形势稳定好转。积极创建广西食品安全示范城市，食品药品安全形势总体平稳。开展扫黑除恶、禁毒“两打两控”和严打整治、打击枪爆违法犯罪、网络犯罪等专项行动，社会治安综合整治不断强化，社会管控工作绩效排名和人民群众对公安机关的满意度都排全区第1位。全力抓好“雪亮工程”、综治中心等信息化建设工作，立体化智能化社会防控体系进一步完善。加快推动军民融合，积极配合做好军队改革转隶、军转干部安置、退伍军人技能培训等工作，推动国防建设和经济建设协调发展。国防后备力量和边防基础设施建设进一步增强，双拥共建工作取得新成果。

　　民族团结进步创建活动深入开展，信访、督查、工商、统计、绩效管理、调处、应急、消防、供销、物价、宗教、侨务、保密、水文、水库移民、邮政、通信、测绘、海关、海事、气象、地震、机关事务等各项工作取得新进展。哲学社会科学、决策咨询、新闻出版、档案、法律援助、妇女儿童、老龄、残疾人等事业取得新进步。工会、共青团、妇联、残联、红十字会、文联、科协、工商联等群团组织在经济社会发展中发挥了重要作用。

　　政府自身建设不断加强。深化领导班子建设年活动，大兴学习之风、调查研究之风、解放思想之风、求真务实之风，建设“五个过硬”领导班子，坚持“两学一做”学习教育常态化制度化。坚持依法行政，完善政府重大决策程序和机制，进一步规范政府权力运行，加强政府立法工作，制定了《百色市公园广场管理办法》。实施“七五”普法工作，提升全民法制意识。推进重点领域政务公开，全年主动公开政府信息超30万条。深入推进反腐倡廉，认真实行廉洁自律准则、纪律处分条例等规定，查处一批违纪违法案件。严格落实中央八项规定精神，推进政府系统开展“四风”整治。审计监督力度进一步增强，实现财政管理流程和财政资金的审计监督全覆盖。全面实施“互联网+政务服务”，大力推动数字百色建设。人大代表议案、建议和政协委员提案办结率100%。

　　二、2019年工作的总体要求和目标任务

　　今年工作的总体要求是：以习近平新时代中国特色社会主义思想为指导，深入贯彻党的十九大和十九届二中、三中全会精神以及庆祝改革开放40周年大会、中央经济工作会议精神，按照“建设壮美广西 共圆复兴梦想”的总目标总要求，统筹推进“五位一体”总体布局，协调推进“四个全面”战略布局，坚持稳中求进工作总基调，坚持新发展理念，坚持推动高质量发展，坚持以供给侧结构性改革为主线，深入开展“六个年”活动，坚持深化市场化改革、扩大高水平开放，继续打好三大攻坚战，推进“三中心两区一市”和左右江革命老区核心城市建设，统筹推进稳增长、促改革、调结构、惠民生、防风险工作，进一步稳就业、稳金融、稳外贸、稳外资、稳投资、稳预期，保持经济持续较快健康发展和社会大局稳定，为决战贫困决胜小康收官打下决定性基础，以优异成绩迎接中华人民共和国成立70周年和百色起义90周年。

　　经济社会发展主要预期目标是：地区生产总值增长9.0%左右，规上工业增加值增长11%，财政收入增长6%，固定资产投资增长10%，外贸进出口总额增长15%，社会消费品零售总额增长10%。城镇人均可支配收入增长6%，农村居民人均可支配收入增长10%。计划实现德保、靖西、凌云、田林4个县（市）脱贫摘帽，约234个贫困村、11.7万贫困人口脱贫。节能减排控制在自治区下达的目标以内。社会各项事业不断进步，社会治理更加现代化，民生福祉持续改善，人民群众获得感幸福感安全感显著增强。

　　（一）以高质量脱贫为使命，坚决打赢脱贫攻坚战。

　　深入推进脱贫攻坚战。严格落实精准扶贫精准脱贫基本方略，抓好产业扶贫、就业扶贫、易地扶贫搬迁、教育扶贫、健康扶贫、生态扶贫、金融扶贫、强化综合保障性扶贫。推动贫困残疾人等特殊人群扶贫工作。大力发展村集体经济，推广资源开发型、资产盘活型、产业带动型等发展模式。聚焦深度贫困地区和极度贫困地区脱贫攻坚，加大对隆林、那坡两个极度贫困县、贫困村、贫困户的支持。严格执行现行扶贫标准，确保完成年度脱贫摘帽任务。

　　打牢可持续脱贫致富基础。加强贫困村基础设施建设，做好资金和项目资源整合，全力推动贫困村屯道路交通、电力电网、广播电视、信息网络、基本公共服务等项目建设。实施大石山区人饮提升改造三年规划，确保在硬件上达到脱贫要求。抓好贫困户子女入学帮扶、健康扶贫“五个一行动”、农村危房改造。加强致富带头人队伍建设，鼓励和引导农村党员带头发展产业，带动群众致富，打造一支“不走的扶贫工作队”。开展扶贫干部大培训。创新激励机制，激发贫困户脱贫内生动力，增强贫困群众脱贫致富能力。加强与深圳在教育、医疗卫生、产业就业等领域协作，加快深圳小镇二期、深百产业园区等项目建设。

　　加强扶贫领域作风建设和实效管理。持续开展扶贫领域腐败和作风问题专项治理，着力查处扶贫领域腐败案件，整治扶贫领域不正之风，压紧压实主体责任。健全工作考核、监督、评估机制，树立鲜明奖惩导向。精简督查考核频次，减轻基层工作负担。

　　（二）以发展先进制造业为重心，推进工业高质量发展。

　　系统推进铝产业高水平发展。加强铝土矿开采的监督和调控，以国家批复氧化铝产能为红线，推动制定矿山中长期采矿规划，严格实行限量开采，延长矿山服务年限，严厉打击非法开采、买卖铝土矿行为。加快调控氧化铝生产，促进氧化铝企业优先保障就地转化，加快实现80%的就地转化率目标。力争百色煤电铝一体化项目全部建成投产，推动电解铝企业实现达产满产，实现电解铝产能237万吨，产量200万吨。建立废铝回收体系，推进百色、新山、平果工业园区等工业区布局再生铝项目，推进福地和平铝80万吨再生铝项目竣工投产，做好铝精深加工招商引资和项目服务工作，力争在铝箔、高纯铝、铝合金导线、全铝汽车、轨道交通等方面取得新进展。推进中兴百色科技产业园、百矿高新铝业、润泰铝业高性能铝板带箔、百兴金兰铝精深加工、中车百矿动车变速箱盖等项目竣工投产。实现铝加工产量超300万吨。做好铝产业配套建设。推进田东60万吨、田林40万吨预焙阳极、铝产品仓储交易中心、天桂铝业一期等配套项目竣工投产。加快废铝、赤泥等产业循环经济和园区循环化改造。

　　加快能源保障基地建设。推进左右江革命老区电力扶贫项目兴义至百色220千伏输变电工程，力争年内完成电源建设。启动建设相关输变电工程，完善和壮大区域电网网架结构。加快推进八渡口水电站建设，提高区域电网的水电比例。发展生物质、光伏、风能发电。保障煤、电、油、气、运等重要生产要素供应。

　　推动优势传统工业振兴。推动石化、冶金、制糖、建材等传统产业转型升级。推动田东石化总厂加快改革发展。以氯碱化工、氟化工为基础，积极拓展下游产业链，发展精细化工。支持百金矿业集团整合开发铜、锰、金等资源，推进百色生态型冶金产业示范基地建设。

　　提升园区发展竞争力。完善新山、百色、平果，田东石化园区等重点园区功能。加大对园区道路、供水、供电、物流等生产性服务设施的升级改造力度，优化公交、学校等园区公共服务设施。优化园区招商环境，加强土地储备与供应，实行弹性用地。加快建设标准厂房、园区科技企业孵化器等平台。加快出台符合不同园区发展的差异性招商引资优惠政策。推进百文园区、桂黔园区、深百产业园等基础设施建设。推进园区招商项目落地，推动百文园区建设50万吨镁铝复合刚玉、复合研磨材料项目，启动中能集团硅电一体化扶贫项目。

　　（三）以发展优势特色产业为核心，统筹实施乡村振兴。

　　推进“10+3”现代特色农业产业。落实粮食安全行政首长责任制。壮大水果、油茶、蔬菜、桑蚕、茶叶、林下养鸡等优势产业，打造休闲农业示范点15个以上、生态有机农业认证面积15万亩以上、富硒农业规模1万亩。开展右江河谷四县（区）生态农业产业带建设。推广丘陵山区农田“宜机化”改造和甘蔗机械化作业。推动一二三产融合发展，重点发展果脯、有机茶、蚕茧、中药材等农产品深加工产业。推动乡村旅游与农业融合发展。重点建设右江、田阳芒果扶贫产业核心示范区，华润五丰生态养殖供港基地、田东国家有机农业综合体、平果布镜湖国际乡村旅游度假区等项目。创建自治区级示范区2个、市级示范区3个、县级示范区4个以上。引进培育一批农业产业龙头企业。

　　增强新型农业经营主体辐射带动能力。加大新型农业经营主体培育力度，新增重点龙头企业5家、农民专业合作社300家、家庭农场80家以上。引导各类新型农业经营主体完善运行机制。搭建农村产权流转交易中心，完成右江、平果、德保、田林、西林农村产权流转交易中心建设。深化与东盟、粤港澳、西南地区等地农业合作，拓展百色农产品销售渠道。

　　提升现代农业品质品牌。严控化肥农药使用量，大力推广“微生物+”生态养殖模式，强化秸秆和畜禽粪污资源化利用。加强农产品质量安全监管，深入创建国家农产品质量安全县。用好“长寿之乡”等资源，打好“绿色生态、长寿百色”品牌。支持鼓励开展农产品地理标志证明商标注册，做大做强百色农产品区域公共品牌、产品品牌，完成10个以上“三品一标”产品认证。

　　实施人居环境整治三年行动。持续推进“美丽百色·幸福乡村”，全面启动乡村风貌提升三年行动，滚动实施一批乡村振兴基础项目。加强农田水利建设、中小河流治理、水土流失治理和新建水源工程建设。落实耕地保护责任制，严格执行“城乡土地增减挂钩”制度，保护永久基本农田。加快实施宽带乡村工程。推进农村“厕所革命”，大力提升村容村貌。做好农业气象灾害监测、预警、服务，提高农业生产防灾减灾能力水平。推进乡风文明建设和乡村治理。

　　（四）以培育新业态新产业为重点，加快发展现代服务业。

　　融合发展文化旅游产业。深化文化和旅游市场改革。推进靖西创建“国家全域旅游示范区”，凌云、田阳等县创建广西特色旅游名县。推进百色起义纪念园、乐业大石围天坑群创建国家AAAAA级景区。加快百色欢乐小镇、福禄河景区、旧州风情小镇等项目建设。开展百色起义90周年红色旅游系列活动。推进历史文化名城创建和壮族文化生态保护区建设。推进“三月三”等传统民俗文化旅游活动，打造“民族风情”旅游品牌。开发红色文化、历史文化、非物质文化遗产旅游产品。建设右江区红色古城、亚洲古人类遗址地质公园等综合文化旅游项目。加快智慧旅游和旅游信息化设施建设。

　　发展健康养生产业。开发长寿养老、度假养生、休闲运动等健康旅游产品，建设百色鼎盛和泰养生文化园、乐业国际长寿养生旅游度假区、靖西国家度假公园等一批综合性度假基地。因地制宜发展板蓝根、八角、灵芝、石斛等特色种植，发展壮药、瑶药等制药企业，建设百东新区生物制药产业园等现代中药原料产业示范区。

　　壮大现代商贸物流业。统筹规划建设新山铝业、平果铝业、百煤物流等现代物流园区。加快中国—东盟农产品交易中心、铝产品仓储交易中心，百色高新区现代服务业基地等项目建设。打造田阳、田东、平果、靖西等区域性物流中心。实现“百色一号”中欧班列每月开行2趟以上。

　　壮大发展电子商务。实施电子商务发展三年行动计划。发展电商扶贫、农商协作等农村电子商务，推进快递网络、物流配送向乡村延伸，建设县、乡、村三级电子商务物流体系和农产品供应链体系、农产品营销体系。创新线上线下融合促销模式，在淘宝网、京东商城等电商平台和企业总部、社区举办“互联网+百色芒果节”。引进跨境电子商务企业落户百色，开展面向东盟的跨境电子商务交流合作和服务。

　　加快数字百色建设。组建百色大数据发展局、设立百色市信息中心。建立数字百色基础项目库，加快实施一批公共服务大数据应用工程项目，在交通物流、医疗卫生、金融服务、城市管理、农业农村等领域应用数字技术。推进数字百色地理空间框架建设，规划建设智慧城市运营管理中心。规划建设数字百色产业园，打造数字产业集聚区。加快与浪潮、科大讯飞、蚂蚁金服、中国联通、和仁科技等互联网科技企业的合作，引进培育一批数字经济新产业、新项目。推动“百企上云”，支持有条件的工业企业进行数字化改造，培育智能车间、智能工厂。实施通信基础设施建设三年行动计划，加快千兆光网城市建设，启动全市5G网络配套资源储备建设。归集全市政务、企业、公众大数据，建设百色数据资源库。

　　（五）以建设美丽宜居城市为目标，打好污染防治攻坚战。

　　扎实开展环境治理。强化大气环境整治，重点治理工业、移动源、扬尘、秸秆焚烧等大气污染源，建成区禁放烟花爆竹。强化水环境整治，实施水污染防治行动计划，全面推行河长制、湖长制，开展集中式饮用水水源地、重点流域水环境综合治理，持续推进下雷河、澄碧河流域、万峰湖库区水环境综合治理，加快建设完善工业园区污水集中处理设施，推进城市黑臭水体、县城内河整治。开展土壤污染状况详查，推进土壤污染风险防控和修复治理项目建设。开展农村生态环境突出问题整治，建设完善农村生活污水、垃圾收集处理设施。严厉打击非法转移、倾倒、处置固体废物的违法犯罪行为。试点开展垃圾回收利用项目。抓好中央环保督察“回头看”反馈意见整改落实。全面开展第二次全国污染源普查。开展环保执法专项行动，全面排查辖区环境问题和风险隐患，推进环境行政执法与刑事司法协调联动。

　　加强生态系统保护和修复。加强生态环境治理，加快推进湿地保护和修复，严守生态环境保护红线，加快实施“山水林田湖草”生态保护与修复工程。持续推进植树造林、封山育林、退耕还林等工程，完成植树造林40万亩，巩固国家森林城市成果。推进气候适应型城市试点建设。加强水土流失、石漠化综合治理，开展矿山地质环境恢复治理，推进国家、自治区级绿色矿山建设。推进国土综合整治工作，加快兴边富民土地整治重大工程建设。强化自然保护区和国家森林公园保护建设。

　　（六）以服务实体经济为着力点，推进金融业稳健发展。围绕金融服务实体经济、防控金融风险、深化金融改革三大任务，进一步优化金融供给，促进经济和金融循环健康发展。

　　加强金融风险防控。全面防范化解企业债务、地方政府债务、房地产市场等重点领域风险。推进交易场所清理整顿和互联网金融风险专项整治，打击非法集资、金融传销等活动。加强信贷资产质量管理和风险监测。加大不良贷款处置力度，探索不良资产证券化“减负”方式。

　　夯实金融业发展基础。继续推动农合行改制组建农商行，推动北部湾银行、右江华润村镇银行等在县域设立金融网点。完善农村金融组织体系，加快培育新型农村金融机构。推进田东金融改革升级和推广。持续推进农村信用“四级联创”工作，创建农村金融“信用市”。完善农村抵押担保体系，稳妥推进农村“两权”抵押贷款试点。持续推动“沿边金改”，主动融入广西建设面向东盟的金融开放门户。开发扶贫信贷新产品，加快发展普惠金融，深化政策性金融扶贫实验示范区建设。

　　创新金融服务实体经济方式。推进金融机构围绕新型产业链和创新链，改进授信评价制度，推出特色信贷产品，打造“一行一品”工程，支持百色铝精深加工、特色农产品加工等产业发展壮大。加大对重点基础设施、保障性安居工程等关键领域的融资支持。深化“银税互动” “政银企担”，加大对民营企业、中小微企业的信贷支持。积极发展消费金融。

　　（七）以落实国家支持百色政策为抓手，提升开发开放水平。

　　推动百色全方位开放。争取国家批准设立广西百色重点开发开放试验区。申报设立百色（靖西）边境经济合作区。推动中国龙邦—越南茶岭跨境经济合作列入“中越跨境经济合作区建设框架协议”。加快推进龙邦口岸升格为国际性口岸、扩大开放至那西通道审批进程。推动龙邦国际商贸物流园升格为国家级综合物流园区。加强与粤港澳大湾区、北部湾经济区、滇黔川渝等地区合作，持续推进跨省区合作园区建设。

　　加快实施左右江革命老区振兴规划。争取国家和自治区加大对百色建设左右江革命老区核心城市支持力度，设立革命老区振兴发展专项资金、新兴产业创新投资基金。筹办全国革命老区振兴论坛。谋划创建国家级经济开发区、综合保税区、承接产业转移示范区等重大国家级平台。争取自治区级边境经济合作区、保税仓库等落地建设。

　　夯实开放开发基础。融入建设西部陆海新通道，加快百色—兴义高速铁路、百色经靖西至防城港高速铁路、云桂沿边（文山—百色—崇左—防城港）铁路、黄桶—百色、靖西—龙邦铁路、南昆铁路百色至威舍扩能改造前期工作。继续开展边境口岸基础设施大会战，启动边境口岸水电网升级改造工程，建立口岸综合应急保障体系。加快右江航道港口码头、作业区建设，畅通右江航道。出台专项优惠政策，促进边境贸易商品落地加工，规划建设进出口贸易加工产业园区。继续推动新兴边民互市点开放运营，促进边境贸易转型升级。发挥万生隆“一站式”口岸综合体等平台作用，加快建成龙邦智慧口岸。

　　积极推进国际产能合作。推动有色金属、农机装备、建材等优势产业走出去，加强与东盟国家经贸合作。推进特色优势产业参与国际开发，建设跨境产业合作园区。利用国内外两种资源、两个市场，建立农产品“东盟生产+中国深加工”产业链。建设国际科技合作基地，推动先进农业新技术、新产品走出去。推动跨境旅游、跨境劳务合作。

（八）以加快百东新区建设为重点，推进新型城镇化建设。

　　加快建设百东新区。推进新区管理体制改革，编制新区经济社会规划发展纲要、控制性详规和城市设计。推进新区基础设施建设攻坚战，完成深百产业园一期路网建设，加快推进新区外域路、星湖路等路网、地下综合管廊建设。完成百色干部学院二校区建设，加快推进右江民族医学院百东校区、百色学院百东校区、百色职业技术学院、百东新区高中项目，全面提升新区教育服务功能。加快推进百色市人民医院、右江民族医学院百东附属医院、桂西生命健康城等医疗项目建设。大力推进深百合作产业园区建设和招商引资，推进深圳小镇二期建设。全面启动百色市行政中心项目申报建设工作。

　　加强城镇基础设施建设。抓好城市路网建设，完善主、次、支路网，加快翔云路、迎龙大道、永乐高速连接线道路和站前大道建设改造。加快东笋片区环境综合整治，改造城市低洼易涝地区，加强公共停车场、城市备用水源、交通慢行道等建设。抓好供电、供气、给排水保障。推进符合条件的多层旧楼加建电梯。均衡增加城市公园绿地，提升森林城市环境质量，做好国家园林城市复查迎检工作。加快推进国家节水城市创建工作，启动国家生态园林城市创建工作。加快县域新客运中心建设。继续推进自治区百镇建设示范工程。

　　实施城市精细化管理。推进文明城市创建，巩固国家卫生城市成果。优化调整城区公交线路，做好百色至田阳客运线路公交化改造工作。加强城市应急体系建设，建立完善城市安全保障工程。整合城市信息资源，构建数字化城市管理系统。扎实开展生活垃圾分类工作。推进海绵城市规划试点建设工作，推进城市建成区违法建设、违法用地专项治理工作。加快扶贫移民和农业转移人口融入城镇，推动落户农村转移人口享受均等化公共服务。完善城镇非户籍人口就业、就医、社保、教育等公共服务政策，推动城镇非户籍人口在城镇落户。

　　拓展城市发展空间。加强右江区、百东新区中心城市功能和辐射带动能力，理顺百东新区、百色高新区、市工业园区、国家农业科技园区、新山工业园区、华侨管理区等新区园区管理机制体制。推进右江河谷城镇带建设，加快田阳撤县设区、平果撤县设市工作。力争城乡建设用地增减挂钩试点项目面积达1.8万亩。抓好平果、靖西国家新型城镇化综合试点和田阳广西新型城镇化示范县建设。力争常住人口城镇化率达到39%。

　　（九）以改善发展环境为突破，促进项目投资大增长。

　　开展“优化营商环境攻坚突破年”活动。深化“放管服”改革。围绕提高效率、降低成本，进一步改善政务、建设经营、融资、税费、人力资源、通关、信用、法治等八大环境，增强企业发展信心与后劲。在百东新区先行先试重大项目建设“五个优化”和投资项目审批“五个简化”。加快建设行政审批“中介超市”。推行审批“容缺受理”机制，全面推广运用投资项目在线并联审批监管平台。推行不动产登记智能化审批。

　　开展“产业招商年”活动。开展大健康、大农业、大物流、新制造、战略性新兴产业五大专题招商。搭建招商引资平台，加强部门联动和政策协调，加快构建“大招商”工作格局，引进一批高质量项目。推动万达、恒大等一批已签约的重大项目落地实施。

　　开展“重大项目建设攻坚突破年”活动。推进市层面重大项目500个以上，力争完成投资400亿元以上，开工项目100个以上，竣工项目60个以上。筹划推进一批新中国成立70周年和百色起义90周年大庆项目。实施好百色水库灌区工程。开通百色至西安航线，推进迁建百色民用机场前期工作，力争开工建设乐业通用机场。建成乐业至百色高速公路，加快建设田林至西林高速公路；推进那坡至平孟、田林至贵州册亨、望谟经乐业至凤山、西林至广南等高速公路前期工作。建成凌云至田林、天生桥至隆林至西林等公路。开工建设田东至田阳、化峒至靖西一级公路，推进平果至田东一级公路等项目前期工作。加快“四好农村路”建设，实施农村公路服务质量提升攻坚工程。加快推进县乡联网公路提级改造，推进乡乡通二级（三级）公路。加快推进田东祥周、田阳头塘、平果旺江及那茂作业区码头工程建设。

　　促进民营经济健康发展。实施强优民营企业培育计划，建立民营经济健康发展机制，大力营造公平竞争政策环境，降低民营企业税费、用人、用地、用电、物流等成本。全面兑现优惠政策承诺，梳理解决“新官不理旧账”等失信行为，全力维护民营企业合法权益。扩大民营经济市场准入，吸引民间资本参与重大项目。拓宽民营企业融资渠道、提升民营企业创新能力、支持民营企业转型发展。构建“亲”“清”新型政商关系，为民营企业家健康成长营造良好的环境。培育壮大民营企业家队伍。更加贴心为民营企业服务，让百色成为民营经济发展的沃土。

　　（十）以高新技术创新应用为支撑，培育驱动发展新引擎。

　　落实创新发展政策。加大科技创新投入，建设多元化投入体系。开展一批产业发展共性关键技术攻关。实施高新技术企业培育发展专项行动，建立高新技术企业培育库，支持企业申报工程技术研究中心、重点实验室，力争全年新入库企业20家以上，新认定高新技术企业3家以上。发挥百色科技创业孵化园、科技企业孵化器等众创空间示范引领作用，为小微企业和个人创新创业提供综合服务。

　　加大产学研融合。实施科技创新支撑产业高质量发展三年行动计划。推进百色高新区升格为国家级高新区。规划建设国家铝金属产品质量监督检验中心二期工程，争取新建院士工作站1个，筹建百色铝产业研究院、铝产业职业技术职教中心、铝产品展示中心、产业协会。在铝产业、冶金、石化、农林产品加工等重点领域新建一批国家级、自治区级工程实验室、工程技术研究中心和企业技术创新中心。全面实施百色人才“百千万工程”，加强国家级专家服务基地建设，引进高校和科研院所高层次人才进驻专家基地。继续实施“蓝火计划”。建立铝产业人才联盟，推动本地企业与区内外高校对接，开展产学研合作，重点培养一批技术带头人和人才团队。

　　全面实施质量强市战略。开展质量提升行动，全面提升产品、工程、服务、产业和人居质量。实施品牌建设工程，创建国家级、自治区级质量品牌，力争参评自治区主席质量奖取得新突破。开展“标准化+”活动，实施农业标准化示范项目，完成广西、百色地方标准的制定。

　　（十一）以深化改革为关键一招，激发经济社会发展新活力。

　　深入推进供给侧结构性改革。组建红城市政发展集团、百金矿业集团、百色一号农业发展集团。着力化解商品非住宅库存。推进电力市场化改革，降低工业企业用电成本。持续降低制度性交易成本。

　　深入推进国资国企改革。完善企业法人治理结构，稳妥推进混合所有制改革，不断优化国有资本布局，深化国有企业内部三项制度改革，推进百矿集团“双百行动”改革。进一步完善企业经营业绩考核和国资监管制度体系建设，确保国有资产保值增值。

　　深化医药卫生体制改革。扩大县域医共体建设范围，提高乡镇卫生院参与率。落实城乡居民大病保险制度，减轻群众看病就医负担。加快建设按病种收付费信息平台，继续推行100个以上病种按病种收费。深入推进药品采购“两票制”，全面推行医用耗材阳光采购和分类采购。

　　扎实推进政府机构改革。调整设置机构，确保上下贯通、执行有力，与中央和自治区改革目标保持一致。结合百色经济社会特点，在限额内设置机构和职能，确保按时完成机构改革任务。

　　深入推进财税体制改革。推进财政事权和支出责任划分改革，推动预算绩效管理向纵深发展。进一步规范政府购买服务。规范政府投资引导基金管理，守住防范风险底线。规范PPP项目运作，推动项目回归公共服务本源。

　　推进农村集体产权制度改革。全面完成农村集体资产清产核资工作。推进集体经营性资产产权制度改革。继续开展集体林地林权证发放查缺补漏，国有林场林地被侵占综合整治等工作。此外，加快推进城市管理执法体制改革。继续深化自治区统计管理体制改革。

　　（十二）以持续增进民生福祉为根本，大力发展社会各项事业。

　　做好就业创业和社会保障。促进农村劳动力特别是贫困劳动力实现转移就业，做好易地扶贫搬迁后续就业创业工作。新增城镇就业1.85万人，农村劳动力转移就业4.6万人次。实施全民参保计划，做到应保尽保。做好社保费移交税务部门征收接续工作。抓好保障农民工工资支付工作。强化对低收入群体的生活兜底保障，加强基层社会救助。加快推进培育和发展住房租赁市场试点工作。棚户区改造2.5万套以上。

　　加快发展教育事业。扩大学前教育资源供给，新增一批公办幼儿园，提高公办幼儿园比例。着力推进县域城乡义务教育均衡发展，力争年内12个县（市、区）全部通过义务教育均衡发展国家评估认定。推进高中阶段教育普及攻坚，抓好32 所普通高中、中职学校新建迁建和改扩建工作。加快发展职业教育，建设一批示范特色专业及实训基地。启动实施“百千万教师培养计划”。加快百色中心城区教育基础设施攻坚项目建设。大力发展高等教育，支持百色高校内涵式发展。

　　扎实推进健康百色建设。制定和完善健康百色政策体系。加强重大传染病防控和卫生应急处置。全面开展县级国家卫生县城创建活动。推进城区医疗卫生项目建设，实现市、县、乡、村卫生计生基础设施建设全面达标。推进基层中医药服务能力提升工程，推广中医、壮瑶医优势病种诊疗技术。加快养老服务业发展，支持公办养老机构公建民营养老。强化食品药品全流程监管，深入开展食品安全科普行动，创建广西食品安全示范城市，确保群众吃上用上放心安全的食品药品。

　　推动文化体育繁荣发展。加快新建或改扩建市、县级三馆和乡镇文化站，推进市县级妇女儿童活动中心、广播电视乡镇无线发射台建设。做好文物和非物质文化遗产保护、文化产业项目申报，打造《扶贫路上》等一批文化精品。承办好广西第十四届运动会。加大公共体育场馆开放力度。大力开展全民健身活动。大力发展体育产业，继续办好“丝路杯”中国—东盟乒乓球赛、中国·百色国际山地户外运动挑战赛、全国徒步大会、红城百色国际半程马拉松赛等重大赛事。

　　加强和创新社会治理。深入推进法治百色、平安百色建设，构建立体化现代化社会治安防控体系。建立健全公共法律服务体系，狠抓执法规范化建设。加强国家安全教育，维护国家安全。持续深入开展扫黑除恶专项斗争，严厉打击涉枪涉爆、毒品犯罪、网络犯罪、非法集资、盗抢骗、传销等违法行为，扎实开展反恐防暴工作，继续深入开展“百里边关无邪教”创建活动。深化信访工作制度改革，推进领导干部接访下访工作的常态化、规范化。深化社会组织管理制度改革，推进城乡社区建设。加强诚信建设。

　　加强应急管理能力建设。健全应急管理体制机制，整合好安全生产、民政救灾、地质灾害、森林防火、公安消防、防汛抗旱、抗震救灾等行业力量，提高综合防灾减灾救灾能力。落实好应急管理责任制，深入开展重点领域隐患排查治理专项活动，坚决防范重特大事故。

　　实施为民办实事工程。千方百计筹措资金，集中力量抓好社保、健康、教育、水利、安居、农补、生态、文化、扶贫、科技等十大类惠民项目。

　　支持部队和国防建设。加强国防教育，加大国防动员、国防后备力量建设力度，继续加强边防基础设施建设和维护。深入开展双拥共建活动，促进军民融合发展。做好退役军人服务管理工作，切实维护军人军属合法权益。

　　办好百色市庆祝新中国成立70周年和纪念百色起义90周年系列活动。加强和改进新形势下民族工作，深入开展民族团结进步创建活动。充分发挥工会、共青团、妇联、残联、红十字会、科协、文联、工商联等群团组织作用。做好志愿服务和社会慈善事业。继续做好宗教、外事、侨务、调处、统计、气象、地震、测绘、人事、编制、档案、老龄、地方志、决策咨询、哲学社会科学等工作。

　　全面提高政府治理能力和治理水平。加强政府系统党的建设。开展“领导班子建设提升年”活动。继续推进“两学一做”学习教育常态化制度化，深入开展“不忘初心、牢记使命”主题教育。严格落实全面从严治党主体责任，严格执行党内法规，深入贯彻落实中央八项规定精神，驰而不息整治“四风”。以更高标准加强党风廉政建设，始终保持反腐败高压态势。加强法治政府建设。严格遵守宪法法律，把政府活动全面纳入法治轨道。落实法治建设第一责任人制度，自觉接受市人大及其常委会的法律监督、工作监督，市政协的民主监督，市监察委的行政监察，认真执行市人大及其常委会决议决定，加强与人大代表、政协委员的沟通联络，办好建议提案。加强审计监督，更加主动地接受全社会监督。坚持依法行政，规范行政执法。发挥好政府法律顾问作用。推进政府决策、执行、管理、服务、结果公开。提升政府行政效能。推动智慧政府建设，建设百色政务云。推进“互联网+政务服务”，推广政务服务“一窗受理、一号申请、一网通办”。优化重构运作流程，精简环节、减少程序，提升审批效率。清理隐性审批和变相审批，强化网上审批、并联审批和集中联审，实现依申请行政权力事项和公共服务事项“最多跑一次”，办税时限压缩三分之一以上的目标。强化工作落实。主动顺应政府机构改革与职能调整转变，优化政府职能配置，构建运行顺畅、令行禁止的工作体系。开展“政策决策学习落实年”活动。弘扬“马上就办、真抓实干、一抓到底”的作风。强化督查督办、考核问责和正向激励，旗帜鲜明为积极干事者撑腰鼓劲，对庸政懒政者严肃问责，促进政府全体工作人员想干事、敢干事、干成事，干出新业绩，干出好口碑。
